# GH receptor polymorphisms guide second-line therapies to prevent acromegaly skeletal fragility: preliminary results of a pilot study

**DOI:** 10.3389/fendo.2024.1414101

**Published:** 2024-08-30

**Authors:** Sabrina Chiloiro, Flavia Costanza, Antonella Giampietro, Amato Infante, Pier Paolo Mattogno, Flavia Angelini, Consolato Gullì, Liverana Lauretti, Mario Rigante, Alessandro Olivi, Laura De Marinis, Francesco Doglietto, Antonio Bianchi, Alfredo Pontecorvi

**Affiliations:** ^1^ Dipartimento di Medicina Traslazionale, Università Cattolica del Sacro Cuore, Rome, Italy; ^2^ Dipartimento di Endocrinologia, Diabetologia e Medicina Interna, Fondazione Policlinico Universitario A. Gemelli, Istituto di Ricovero e cura a carattere scientifico (IRCCS), Rome, Italy; ^3^ Dipartimento di Diagnostica per Immagini e radioterapia oncologica, Fondazione Policlinico Universitario A. Gemelli, Istituto di Ricovero e cura a carattere scientifico (IRCCS), Rome, Italy; ^4^ Dipartimento di Neurochirugia, Fondazione Policlinico Universitario A. Gemelli Istituto di Ricovero e cura a carattere scientifico (IRCCS), Rome, Italy; ^5^ Dipartimento di Science dell’invecchiamento, neuroscienze, delle scienze del capo-collo, ed ortopediche, Università Cattolica del Sacro Cuore, Rome, Italy; ^6^ Unità di Otorinologingoiatria, Fondazione Policlinico Universitario A. Gemelli Istituto di Ricovero e cura a carattere scientifico (IRCCS), Rome, Italy

**Keywords:** fracture, osteopenia, osteoporosis, acromegaly, pegvisomant, somatostatin receptor ligands, pasireotide, GH receptor

## Abstract

**Background:**

Skeletal fragility is characterized by increased frequency of vertebral fractures (VFs) in acromegaly. Several trials were conducted to identify modifiable risk factors and predictors of VFs, with limited data on the prognostic role of GH receptor (GHR) isoforms. In this study, we investigated the potential role of GHR polymorphism on the occurrence of incidental VFs (i-VFs), in patients treated with second-line medical therapies.

**Methods:**

A longitudinal, retrospective, observational study was conducted on a cohort of 45 acromegalic patients not-responsive to first-generation somatostatin receptor ligands (fg-SRLs) and treated with GHR antagonist (Pegvisomant) or with the second-generation SRLs (Pasireotide long-acting release).

**Results:**

Second line treatments were Pegvisomant plus fg-SRLs in 26 patients and Pasireotide LAR in 19 patients. From the group treated with fg-SRLs+Peg-V, the fl-GHR isoform was identified in 18 patients (69.2%) and the d3-GHR isoform in 8 patients (30.8%). I-VFs arose exclusively in fl-GHR isoform carriers (p=0.039). From the group treated with Pasireotide LAR, the fl-GHR isoform was identified in 11 patients (57.9%), and the d3-GHR isoform in 8 patients (42.1%). I-VFs arose exclusively in d3-GHR isoform carriers (p=0.018). Patients with fl-GHR isoform had a higher risk for i-VFs if treated with fg-SRL+Peg-V (OR: 1.6 95%IC: 1.1-2.3, p=0.04), and a lower risk if treated with Pasi-LAR (OR: 0.26 IC95%: 0.11-0.66, p=0.038).

**Conclusions:**

Our data support a predictive role of the GHR isoforms for the occurrence of i-VFs in acromegalic patients treated with second-line drugs, tailored to the individual patient. The knowledge of the GHR polymorphism may facilitate the choice of second-line therapies, improving the therapeutic approach, in the context of personalized medicine.

## 1 Introduction

Acromegaly is a systemic disease, characterized by an autonomous overproduction of the growth hormone (GH) and of the insulin growth factor I (IGF-I), which can modulate and regulate bone metabolism (1), increasing bone turnover with the subsequent deterioration of cortical and trabecular bone structures, affecting bone quality and quantity and therefore increased risk of vertebral fractures (VFs) (2). Musculoskeletal disorders are emerging and not negligible acromegaly-related complications, involving up to 25–40% of patients (1–6), affecting the quality of life (QoL), in acromegalic patients (1). The risk of fractures is not completely normalized by the achievement of the biochemical control of acromegaly (7). The management of acromegaly-related skeletal fragility is still challenging since the prediction of VFs is uncertain. The higher levels of GH and IGF-I, the longer duration of active disease, the presence of pre-existing VFs and hypogonadism, the higher daily substitutive doses of hydrocortisone (or equivalent), and the absence of vitamin D supplementation, and diabetes mellitus were identified as the most relevant risk factors for skeletal fragility (2, 4, 5, 8–11).

The prevention of VFs in acromegaly remains an unresolved issue. The administration of vitamin D supplementation and bone active drugs may reduce the risk of the development of VFs (8, 12–15). In parallel, the treatment with GH/IGF-I lowering therapies (such as fg-SRLs, Pegvisomant and Pasireotide Lar) was proved to decrease the frequency of i-VFs in acromegaly through an indirect effect on bone metabolism, mediated by the reduction/normalization of circulating levels of IGF-I and/or GH (14, 15). The potential direct effects of GH/IGF-I lowering drugs on bone metabolism have not been fully clarified. A recent study proved that octreotide may inhibit murine primary osteoblasts and osteoblast cells proliferation through the action of somatostatin receptors (16). A subsequent study suggested that Pegvisomant may modulate osteoblast cell proliferation, differentiation, and mineralization, through GH action, rather than by a direct action of Pegvisomant on the osteoblast cell metabolism (17).

This study aims to deepen our knowledge of the genetic and cellular mechanisms involved in bone metabolism in acromegaly, by examining the potential predictive value of the GH receptor (GHR) isoforms on the occurrence of i-VFs. Until now, two isoforms of the GHR were reported: the wild-type or full-length form (fl-GHR), and the mutated/deleted isoform, which is characterized by the failure of the exon 3 transcription (d3-GHR). The exon 3 encodes a portion of the extracellular domain of the GHR (18–20), and its presence or absence results in a different affinity of its ligand: the GH (21). Therefore, the potential effect of GHR polymorphism on growth, height, weight, body composition, glucose, and lipid metabolism was investigated in several diseases, involving the secretion of GH and or IGF-I, such as the Prader-Willi syndrome, Turner syndrome, small for gestational age, and growth hormone deficiency (18, 22). Since then, the correlation between different GHR genotypes and systemic comorbidities in acromegalic patients has been examined by numerous studies, that were mainly focused on hypertension, obesity, diabetes mellitus type 2, colonic polyps, heart disease, obstructive sleep apnea syndrome, bone fragility, and heart disease (23–28), without reaching univocal findings. In acromegaly, the patients carrying the d3-GHR isoform showed a better clinical response to therapy with Pegvisomant (18, 24). Some studies have demonstrated that that acromegalic patients carrying the d3-GHR isoform had an increased prevalence of VFs, with respect to fl-GHR carriers (21).

To our knowledge, no studies have been designed and published until now to investigate the prognostic role of the GH receptor isoforms according to the different classes of medical treatments in acromegaly.

The primary objective of this pilot study was to compare the frequency of i-VFs according to the different GH receptor polymorphisms, in two groups of patients treated with second-line medical therapies: Pasireotide LAR or fg-SRLs plus Pegvisomant. As secondary objectives, we investigated the correlation between the occurrence of incidental vertebral fractures (i-VFs) and other known relevant risk factors for VFs such as gender, age at the diagnosis of acromegaly, serum GH and IGF-I level (at diagnosis, at the start of second-line therapies and at last follow-up), prevalent vertebral fractures (p-VFs), hypopituitarism, and doses of substitutive hydrocortisone (or equivalent), glucose metabolism, use of vitamin D supplementation and bone active drugs, in both groups of treatment.

## 2 Materials and methods

Study design: longitudinal, retrospective, observational, and monocenter study

Study population: Patients with acromegaly were consecutively included according to the following criteria.

The inclusion criteria were:

1. patients with active disease (with IGF-I values over the normal age-adjusted reference ranges, random GH of more than 1.0 mcgr/L and/or regrowth of tumor remnant) (15), after at least 6 consecutive months of therapy with first-generation somatostatin receptor ligands (fg-SRLs) at standard dose, followed by further 6 consecutive months of increased dose frequency (Lanreotide ATG 120 mg every 21 days or Octreotide Lar 30 mg every 21 days) (16); and consequently treated with second line medical therapies (Pasireotide Lar or Pegvisomant in association with fg-SRLs), for at least 12 consecutive months;

2. patients > 18 years;

3. availability of study-related data;

4. at least 12 months follow-up, since the study entry.

We excluded from the study patients with active neoplasia, primary hyperparathyroidism and MEN-1 syndrome, untreated hyperthyroidism, previous or current treatment with drugs known to cause fragility fractures (17), except glucocorticoid replacement therapy for central adrenal insufficiency and history of spine surgery or trauma.

### 2.1 Study protocol

Due to the study’s retrospective design, the baseline visit corresponded to the day of the clinical visit with the prescription of second-line medical therapies: Pegvisomant (Peg-V) or Pasireotide Lar (Pasi-Lar). The treatment choice for Peg-V or Pasi-LAR was based on the physician’s clinical judgment and on the last expert consensus (29), as also reported in our previous study (30).

According to our clinical practice, patients with acromegaly on treatment with second-line medical therapies were clinically and biochemically evaluated at least every six months. Dose titrations for Peg-V and Pasi-LAR were performed according to clinical practice, considering disease related signs and symptoms, levels of IGF-I and GH (when appropriate), residual tumor shrinkage, treatment safety, tolerance, and compliance. According to our clinical practice and to 2013 guidelines on the management of acromegaly-related comorbidities (32), X-rays of thoracic and lumbar spine were conducted at fixed time points: at one year of follow-up since the start of treatment with Peg-V and Pasi-LAR, and then annually in patients with clinical and biochemical active acromegaly, being considered at higher risk for further deterioration of bone health. I-VFs were investigated at 2-years thoracic and lumbar spine X-rays in patients who had reached the biochemical control of disease (controlled patients). The conclusion of the follow-up coincided with the final visit and the availability of all clinical data pertaining to acromegaly and bone health, as required by the study protocol, after a minimum of twelve consecutive months of treatment. At the conclusion of the study, patients were deemed controlled if their IGF-I values were within the reference ranges for age (at least in two consecutive measures) and their random GH was below 1.0 mcgr/L (31). Patients receiving treatment with Peg-V were evaluated only using serum IGF-I. The IGF-I was expressed as absolute values and as IGF-I for the upper limit of normality.

### 2.2 Data collection

For the baseline visit, the following data were collected: gender, age and levels of both GH and IGF-I at acromegaly diagnosis and at baseline, previous treatments for acromegaly, concomitant hypopituitarism, gonadal function, diagnosis of prevalent VFs (p-VFs), concomitant medications (including the use of replacement therapy for hypopituitarism), choice of second-line therapy with fg-SRLS plus Peg-V or Pasi-LAR.

At last follow-up, we collected data on serum GH and IGF-I levels, gonadal function, hypopituitarism, use of replacement therapy for central adrenal insufficiency and dosage, of vitamin D supplementations and bone active drugs and occurrence of i-VFs.

### 2.3 Evaluation of vertebral fractures

VFs were investigated through a semiquantitative morphometric approach (32). The height of the anterior, middle and portions of each vertebra (respectively Ha, Hm and Hp) was measured on vertebral morphometry of dorso-lumbar spine x-ray. From each vertebra (from T4 to L4) the ratio of the heights of each vertebral portion was calculated (Ha/Hp, Hm/Hp or Hp/Hp), and according to Genant’s classification (33), VFs were defined in cases of ratio decrease >20%. P-VFs were identified on the basal radiographs, whereas i-VFs were identified on spinal radiographs obtained during follow-up but absent at baseline.

### 2.4 GHR isoform assessment

The GHR genotype (flfl, fld3, or d3d3) was determined on genomic DNA that was extracted from 100/200 μ/L peripheral blood. Polymorphisms were studied through polymerase chain reaction (PCR), as previously reported (34). Electrophoresis separated the amplification products from each other by separation. The full-length allele (fl-GHR) was identified as a 935 bp fragment and the exon 3-deleted allele (d3-GHR) by a 532 bp fragment.

### 2.5 Statistical analysis

Descriptive statistical methods were employed to describe the patient group’s clinical and demographic characteristics. Kolmogorov-Smirnov test was used to check the normality of continuous variables. The chi square test (or Fisher exact test when necessary) and Mann Whitney non-parametric tests were used to compare categorical and quantitative un-paired data. Age at treatment, gender and risk factors found to have a p <0.25 at univariate analysis were included in the multivariate logistic regression. Only cases with complete data were used for the analysis (complete case analysis), as specified in inclusion and exclusion criteria. As the aim of this prediction study is the identification of a specific outcome (occurrence of p-VFs) through the combination of all predictors in the model, specific confounders were not singled out. SPSS software version 24.0 for Windows was used for the statistical investigations.

### 2.6 Ethical approval

All procedures performed in the study were in accordance with the ethical standards of the institutional review board and with the 1964 Helsinki declaration and its later amendments or comparable ethical standards. The study was approved by local Institutional Review Boards. All patients signed an informed consent before entering the study.

## 3 Results

Forty-five acromegalic patients entered the study. Females were thirty-one (68.9%). The age at acromegaly diagnosis was 39 years (IQR: 17). Median GH levels at acromegaly diagnosis was 20 ng/mL (IQR: 26), IGF-I was 892 ng/mL (IQR: 214) and IGF-I x ULN was 2.7 (IQR: 1.8).

Sixteen patients were d3-GHR isoform carriers (35.6%), and twenty-nine were fl-GHR isoform carriers (64.4%).

At study entry, acromegaly was clinical and biochemical active in all included patients, requiring second-line therapies median GH: 4.5 ng/mL IQR: 4.2, IGF-I: 371 ng/mL IQR: 372, IGF-I x ULN: 1.75 IQR:1.5. Age of included patients at baseline was 45.7 years IQR:12. All patients had undergone pituitary surgery and 12 consecutive months of treatment with fg-SRLs. Nine patients carried p-VFs (20%). Nineteen patients were affected by secondary hypoadrenalism (42.2%); ten patients were affected by secondary hypogonadism (22.2%). Twenty-six patients were treated with fg-SRLs plus Peg-V (57.8%) and nineteen were treated with Pasi-LAR (42.2%). Patients treated with fg-SRLs plus Peg-V and those treated with Pasi-LAR did not differ for gender, age, GH and IGF-I levels at acromegaly diagnosis, at baseline and at the last follow-up, concomitant hypopituitarism, GHR polymorphisms, frequency of p-VFs, and acromegaly outcome, as detailed in **Table 1**.

**Table 1:** Clinical, hormonal, and genetic features in study population, also stratified for treatment groups.

	Whole study population	Fg-SRLs plus Peg-v treated group	Pasireotide Lar treated group	p-value
Gender Females n, (%) Males n, (%)	31 (68.9%) 14 (31.1%)	17 (65.4%) 9 (34.6%)	14 (73.7%) 5 (26.3%)	0.553
Age at baseline, years median (IQR)	45.7 (12)	42.8 (26.5)	42 (17.9)	0.267
GH baseline ng/mL median (IQR)	4.5 (4.2)	7.4 (22)	5.3 (9.1)	0.383
IGF-I at baseline ng/mL median, (IQR) ULN median, (IQR)	371 (372) 1.75 (1.5)	574 (287) 2 (1.2)	353 (302) 1.1 (1.1)	0.04 0.014
Secondary hypogonadism No n, (%) Yes n, (%)	35 (77.8%) 10 (22.2%)	18 (69.2%) 8 (30.8%)	17 (89.5%) 2 (10.5%)	0.104
Secondary hypoadrenalism No n, (%) Yes n, (%)	26 (57.8%) 19 (42.2%)	16 (61.5%) 10 (38.5%)	10 (52.6%) 9 (47.4%)	0.385
Hydrocortisone or equivalent dose ≤ 20 mg/daily > 20 mg/daily	5 (26.3%) 14 (73.7%)	3 (30%) 7 (70%)	2 (22.2%) 7 (77.8%)	0.556
GHR polymorphism d3-carriers n, (%) fl-carries n, (%)	16 (35.6%) 29 (64.4%)	8 (30.8%) 18 (69.2%)	8 (42.1%) 11 (57.9%)	0.433
Prevalent VFs No n, (%) Yes n, (%)	36 (80%) 9 (20%)	21 (80.8%) 5 (19.2%)	15 (78.9%) 4 (21.1%)	0.88
Acromegaly disease at follow-up Controlled n, (%) Active n, (%)	38 (84.4%) 7 (15.6%)	20 (76.9%) 6 (23.1%)	18 (94.7%) 1 (5.3%)	0.103
Follow-up months median, (IQR)	57.1 (28.6)	59.4 (29.9)	52.4 (26.2)	0.48
GH ng/mL at follow-up median, (IQR)	Na	Na	0.7 (1.1)	Na
IGF-I at follow-up ng/mL median, (IQR) ULN median, (IQR)	178 (88) 0.7 (0.3)	176 (152) 0.8 (0.7)	155 (73) 0.5 (0.3)	0.196 0.026
Glucose metabolism at follow-up Normal n, (%) IGT/DM2 n, (%)	5 (11.1%) 40 (88.9%)	3 (11.5%) 23 (88.5%)	2 (10.5%) 17 (89.5%)	0.915
Vitamin D supplementation Treated patients n, (%) Not-treated patients n, (%)	32 (71.1%) 13 (28.9%)	18 (69.2%) 8 (30.8%)	14 (737%) 5 (26.3%)	0.506
Bone active drugs Treated patients n, (%) Not-treated patients n, (%)	6 (13.3%) 39 (86.7%)	4 (15.4%) 22 (84.6%)	2 (10.5%) 17 (89.5%)	0.496

Univariate analysis. Data are presented as n (%) or as median (IQR).

At the last follow-up, thirty-eight patients were considered controlled for acromegaly (84.4%), and seven patients were affected by active disease (15.6%). Eleven patients experienced the occurrence of i-VFs (24.4%). Median follow-up was 57.1 months (IQR: 28.6).

### 3.1 Pegvisomant plus first-generation SRLs treatment group

Eighteen patients out of the 36 included in this treatment group were fl-GHR isoform carriers (69.2%) and eight patients were d3-GHR isoform carriers (30.8%). At baseline, the Median age at baseline was 42.8 IQR:26.5, GH was 7.4 ng/mL (IQR:22), IGF-I was 574 ng/mL (IQR: 287) and IGF-I x ULN was 2 (IQR:1.2). Five patients carried p-VFs (19.2%).

At the last follow-up, the mean dosage of Peg-V was 15 mg/daily (range: 10-40 mg/daily); six patients were considered affected by active disease (23.1%).

I-VFs were identified in seven patients, at the last follow-up (26.9%). Clinical and molecular features of patients treated with Pasi-LAR, according to the occurrence of i-VFs at follow-up were summarized in **Table 2**. The occurrence of I-VFs did not differ for gender, age, GH and IGF-I levels (tested at acromegaly diagnosis, at baseline, and at the last follow-up), concomitant hypopituitarism, frequency of p-VFs, acromegaly outcome and glucose metabolism at follow-up. All patients who developed i-VFs carried the fl-GHR isoform (100%); no d3-GHR isoform carriers experienced i-VFs (0%, p=0.039). Among the nineteen patients who did not develop i-VFs, eight were d3-GHR isoform carriers (42.1%) and eleven were fl-GHR isoform carriers (57.9%, p=0.039).

**Table 2:** Clinical, hormonal, and genetic determinants of i-VFs in patients treated with first generation SRLs + pegvisomant.

	Fg-SRLs + Peg-V treatment group			Pasireotide Lar treated patients		
	Incidental VFs			Incidental VFs		
	No	Yes	p-value	No	Yes	p-value
Gender Females n, (%) Males n, (%)	13 (68.4%) 6 (31.6%)	4 (57.1%) 3 (42.9%)	0.592	11 (73.3%) 4 (26.7%)	3 (75%) 1 (25%)	0.728
Age at baseline, years median (IQR)	43.5 (24.2)	40.1 (39.3)	0.804	45.7 (15.4)	39.6 (15.8)	0.953
GH baseline ng/mL, median (IQR)	12.7 (23)	13.6 (10.6)	0.4	4.5 (6.7)	3.7 (7)	0.808
IGF-I at baseline ng/mL, median (IQR) ULN, median (IQR)	582 (382) 2.2 (1.7)	394 (310) 1.7 (1.1)	0.193 0.069	625 (486) 2.2 (1.7)	197 (263) 1.1 (0.6)	0.154 0.26
Secondary hypogonadism No n, (%) Yes n, (%)	15 (78.9%) 4 (21.1%)	3 (42.9%) 4 (57.1%)	0.149	14 (93.3%) 1 (6.7%)	3 (75%) 1 (25%)	0.386
Secondary hypoadrenalism No n, (%) Yes n, (%)	12 (63.2%) 7 (36.8%)	4 (57.1%) 3 (42.9%)	0.562	8 (53.3%) 7 (46.7%)	2 (50%) 2 (50%)	0.667
Hydrocortisone or equivalent dose ≤ 20 mg/daily > 20 mg/daily	2 (28.6%) 5 (71.4%)	1 (33.3%) 2 (66.7%)	0.587	2 (28.6%) 5 (71.4%)	0 (0%) 2 (100%)	0.587
GHR polymorphism d3-carriers n, (%) fl-carries n, (%)	8 (42.1%) 11 (57.9%)	0 (0%) 7 (100%)	0.039	4 (26.7%) 11 (73.3%)	4 (100%) 0 (0%)	0.018
Prevalent VFs No n, (%) Yes n, (%)	17 (89.5%) 2 (10.5%)	4 (57.1%) 3 (42.9%)	0.1	13 (86.7%) 2 (13.3%)	2 (50%) 2 (50%)	0.178
Acromegaly disease at FUP Controlled n, (%) Active n, (%)	14 (73.7%) 5 (26.3%)	6 (85.7%) 1 (14.3%)	0.471	15 (100%) 0 (0%)	3 (75%) 1 (25%)	0.211
GH at follow-up median (IQR)	Na	Na	Na	1.4 (4.3)	0.4 (0.4)	0.178
IGF-I Follow-up ng/mL, median (IQR) ULN, median (IQR)	178 (283) 0.8 (1)	176 (105) 0.7 (0.1)	0.579 0.71	125 (87) 0.5 (0.3)	193 (30) 0.7 (0.2)	0.03 0.048
Glucose metabolism at follow-up Normal n, (%) IGT/DM2 n, (%)	2 (10.5%) 17 (89.5%)	1 (14.3%) 6 (85.7%)	0.627	0 (0%) 15 (100%)	2 (50%) 2 (50%)	0.35
Vitamin D supplementation Treated patients n, (%) Not-treated patients n, (%)	14 (73.7%) 5 (26.3%)	4 (57.1%) 3 (49.2%)	0.361	13 (86.7%) 2 (13.3%)	1 (25%) 3 (75%)	0.037
Bone active drugs Treated patients n, (%) Not-treated patients n, (%)	3 (15.8%) 16 (84.2%)	1 (14.3%) 6 (85.7%)	0.713	2 (13.3%) 13 (86.7%)	0 (0%) 4 (100%)	0.614

Univariate analysis. Data are presented as n (%) or as median (IQR).

### 3.2 Pasireotide LAR treatment group

Eleven patients out of the 19 included in this treatment group were fl-GHR isoform carriers (57.9%) and eight patients were d3-GHR isoform carriers (42.1%). Median age at baseline was 42 years (IQR:17.9), GH was 5.3 ng/mL (9.1), IGF-I was353 ng/mL IQR: 302 and IGF-I x ULN 1.1 (IQR:1.1). At baseline, p-VFs occurred in 4 patients (21.1%). A single patient was considered affected by active acromegaly (5.3%) at last follow-up.

Clinical and molecular features of patients treated with Pasi-LAR, according to the occurrence of i-VFs at follow-up were summarized in **Table 2**. I-VFs occurred exclusively d3-GHR isoform carriers (100%), with respect of fl-GHR isoform carriers (0%, p=0.018). Among the fifteen patients who did not develop i-VFs, four were d3-GHR isoform carriers (26.7%) and eleven were fl-GHR isoform carriers (73.3%). I-VFs occurred more frequently in patients with higher IGF-I levels at the last evaluation (median IGF-I: 193 ng/mL IQR: 30 p=0.03, median IGF-I x ULN: 0.7, IQR:0.2, p=0.048), as compared to patients without i-VFs (mean IGF-I: 125 ng/mL IQR: 87, median IGF-I x ULN: 0.5 IQR: 0.3). I-VFs were significantly less frequent in patients treated with vitamin D supplementation. Frequency of i-VFs was superimposable in patients with normal glucose metabolism and with glucose intolerance or diabetes mellitus, in both treatment groups, as reported in **Table 2**.

### 3.3 GHR polymorphism in treatment groups

Gender, GH and IGF-I levels at baseline, p-VFs frequency, acromegaly outcome, GH and IGF-I levels at the last follow-up did not differ among patients carrying the d3-GHR or fl-GHR isoforms, in fg-SRLs plus Peg-V treatment group (**Table 3**).

**Table 3:** Clinical, hormonal, and bone metabolism according to GHR polymorphisms in patients treated with fg-SRLs + Peg-V and Pasireotide Lar.

	Fg-SRLs + Peg-V treatment group			Pasireotide Lar treatment group		
	d3-GHR carriers	Fl-GHR carriers	p-value	d3-GHR carriers	Fl-GHR carriers	p-value
Gender Females n, (%) Males n, (%)	5 (62.5%) 3 (37.5%)	12 (66.7%) 6 (33.3%)	0.587	7 (87.5%) 1 (12.5%)	7 (63.6%) 4 (36.4%)	0.267
Age at baseline, years median (IQR)	66.7 (22.3)	40.1 (13.5)	0.009	37.8 (23.4)	42 (19.5)	0.279
GH at baseline ng/mL, median (IQR)	11.4 (23)	12.7 (27.4)	0.998	4 (8.5)	6.6 (10.1)	0.394
IGF-I at baseline ng/mL, median (IQR) ULN, median (IQR)	574 (788) 2.8 (3.5)	554 (281) 2 (1)	0.506 0.095	321 (96) 0.98 (0.33)	564 (553) 1.9 (1.5)	0.106 0.073
Prevalent VFs No n, (%) Yes n, (%)	7 (87.5%) 1 (12.5%)	14 (77.8%) 4 (22.2%)	0. 502	6 (75%) 2 (25%)	9 (81.8%) 2 (18.2%)	0.574
Acromegaly disease at follow-up Controlled n, (%) Active n, (%)	5 (62.5%) 3 (37.5%)	15 (83.3%) 3 (16.7%)	0.249	7 (87.5%) 1 (12.5%)	11 (100%) 0 (0%)	0.421
GH ng/mL, median (IQR) at follow-up	Na	Na	Na	0.55 (2.3)	1.1 (2.6)	0.61
IGF-I Follow-up ng/mL, median (IQR) ULN, median (IQR)	195 (324) 1 (1.4)	172 (146) 0.7 (0.5)	0.535 0.166	178 (45) 0.7 (0.3)	125 (46) 0.5 (0.12)	0.038 0.232

Univariate analysis. Data are presented as n (%) or as median (IQR).

Among the group of patients treated with Pasi-LAR, IGF-I levels were higher in d3-GRH carriers than in fl-GHR carriers, at the last follow-up (p=0.038), despite GH levels being similar (respectively 0.55 ng/mL IQR: 2.3 in d3-GRH carriers and 1.1 ng/mL IQR: 2.6 in fl-GHR carriers, p=0.61). Moreover, at baseline both GH and IGF-I serum concentrations were superimposable among d3-GRH and fl-GHR carriers, as detailed in **Table 3**.

### 3.4 Logistic regression

**Figure 1:**
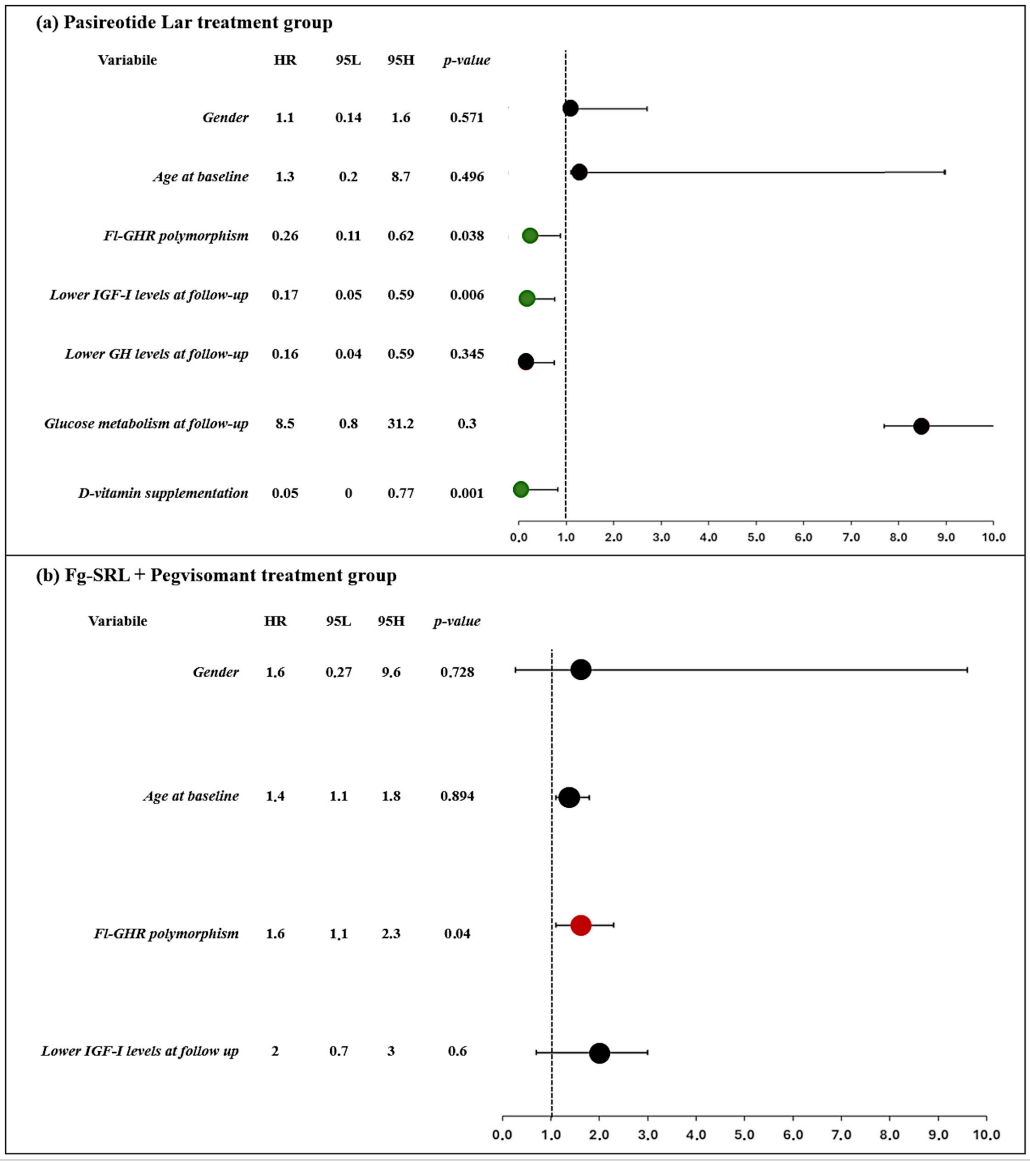
Clinical and molecular predictors of occurrence of incidental VFs in patients treated with Pasireotide Lar **(A)** and first generation SRLs + Pegvisomant **(B)**. Forrest plot represents the odds ratio.

As shown in **Figures 1A, B**, patients carrying the fl-GHR isoform had a higher risk for i-VFs if treated with Peg-V, while showing a lower risk of i-VFs if treated with Pasi-LAR. The risk of i-VFs was reduced in individuals with lower IGF-I levels at the last observation during treatment with Pasi-LAR. The vitamin D supplementation was protective from the occurrence of i-VFs in patients treated with Pasireotide Lar.

## 4 Discussion

In this pilot study we explored the potential GHR isoform’s prognostic role in the development of i-VFs in acromegalic patients treated with GH receptor antagonist and with Pasi-LAR. In this cohort of patients, we found that all patients who experienced i-VFs during the treatment with Peg-V carried the fl-GHR isoform and all patients which experienced i-VFs during the treatment with Pasi-LAR carried the d3-GHR isoform.

These results were consistent with previously published data on GHR polymorphisms in predicting the biochemical outcome of treatments with Peg-V and Pasi-LAR. Acromegalic patients carrying the d3-GHR were reported to require a lower Peg-V dose and a shorter treatment duration to normalize IGF-I levels, at least during the first year of treatment (18). This protective d3-GHR effect seems to weaken as the treatment duration is prolonged, possibly due to the progressive saturation of the GHR (18). In fact, a 4-year predictive study conducted by our research team (21) and a recent meta-analysis of Franck et al. (35) ruled out a long-term GHR isoform effect on Peg-V efficacy. None data are instead available on the possible down-regulation of the GHR expression during treatment with Peg-V.

Data on the possible effects of the GHR isoforms on the outcome of treatment with Pasi-LAR are limited to our recent experience conducted on 33 acromegalic patients, showing that d3-GHR carriers were more frequently not able to reach biochemical control during Pasi-LAR treatment, and that fl-GHR carriers have been good Pasi-LAR responders, despite similar values of GH and IGF-I before starting Pasi-LAR treatment (21). The positive effects in lowering IGF-I secretion of d3-GHR isoform in Peg-V treated patients and of fl-GHR isoform in Pasi-LAR treated patients may therefore induce a better biochemical control of acromegaly and in parallel may reduce the risk of the occurrence of VFs, which recognize among the main risk factors the persistence of high IGF-I levels. Despite the inability of our data to definitively elucidate the underlying molecular pathway, an interaction between drugs, GH, GHR and its polymorphisms is inferred by considering that the IGF-I levels were higher in d3-GHR isoform carriers, with respect to fl-GHR isoform carriers, despite overlapping GH levels, both at baseline and at follow-up, in patients treated with Pasi-LAR. Patients carrying the fl-GHR isoform on treatment with Pasi-LAR may experience i-VFs less frequently, because fl-GHR is “*per-se*” less sensitive to GH stimulation, therefore reducing the activation of intracellular second messengers and consequently reducing the gene transcription and protein synthesis, finally resulting in lower serum concentrations of IGF-I. This finding was also confirmed by the higher IGF-I levels in patients who experienced p-VFs. In parallel, no data are available the expression of the somatostatin receptor on bone in human. Therefore, a possible direct effect of Pasireotide Lar through the somatostatin receptors can only be hypothesized to date. Recently, in fact, the expression of subtypes 2 and 5 of somatostatin receptors has been demonstrated on cell cultures of murine osteoblasts and pre-osteoblasts (16)

Our results also support a possible different effect of GHR isoform in patients treated with Pegvisomant. The data of our cohort support a protective role of the GHR polymorphisms, also independently of the reaching of biochemical control of acromegaly, and independently from the final IGF-I levels, supporting the hypothesis that the interaction of GHR polymorphism and treatment modality in fracture risk is independent of treatment outcome, as reported in **Figure 1**. Although the underlying molecular mechanisms remain still not definitively understood, it was previously assumed that the d3-GHR have an increased affinity for the pegvisomant and the native GH, increased dimerization capacity and consequently enhanced intracellular signal transduction (29). It was hypothesized that blocking a receptor with such improved functional properties would result in an amplification of the inhibitory action of Peg-V. Furthermore, differences in the ability to internalize Peg-V could also be responsible for these findings. These hypothesized molecular mechanisms may justify the lower frequency of i-VFs in d3-GHR acromegaly carriers, treated with Peg-V, rather than to fl-GHR carrying ones.

The retrospective design and the small cohort size of the study population are the main limitations of our study. The restricted number of included patients reflects the stringent inclusion criteria and the rarity of the acromegaly disease, as well as the selection of a consecutive cohort of acromegalic patients resistant to fg-SRLs and treated with second-line therapies, Pegvisomant and Pasireotide Lar, to examine the interactions of GHR, bone health and GH/IGF lowering therapies in a cohort of acromegalic patients affected from a more difficult/aggressive disease.

Moreover, the absence of data of circulating levels of GH and pegvisomant, during the treatment with the GHR antagonist did not allow us the definitively clarify the mechanisms of interaction of GH, Peg-V and different GHR isoforms. As reported in acromegaly guidelines (36), the dosage of GH is not recommended in patients treated with Pegvisomant to define the biochemical control for the expected increase of GH levels due to the negative-feedback loop (37).

Therefore, additional studies are advocated to ascertain and validate our findings on larger populations, with a randomized and prospective design, also including data deriving from innovative tools to evaluate bone health, such as high-resolution peripheral quantitative computed tomography (HR-pQCT) and the trabecular bone score (TBS), that are actually recognized to be very useful to assess bone microarchitecture and predict the fracture risk in acromegaly (38–40).

Our study proved for the first time that the GHR polymorphisms may orient the choice of second-line medical therapies in acromegaly, considering the prevention of acromegaly-related comorbidities, such as bone health. According to our results, we may speculate that the GHR isoform may be integrated to clinical, genetic, and molecular and morphological tumor biomarkers, for an individual patient tailored therapy. A higher chance of GH and IGF-I normalization and a lower risk/frequency of i-VFs are associated with the fl-GHR isoform in patients treated with Pasi-LAR. In parallel, d3-GHR isoform is associated with a greater change of GH and IGF-I normalization at least at one year of treatment with Peg-V and with a lower risk/frequency of i-VFs.

In conclusion, although our results should be considered preliminary, our findings support that the investigation of the GHR polymorphism may prove beneficial in enhancing the personalized therapeutic approach of patients with acromegaly, including the prevention of acromegaly-related comorbidities, in the context of patients-tailored medicine.

## Data Availability

The raw data supporting the conclusions of this article will be made available by the authors, without undue reservation.

## References

[1] Giustina A Mazziotti G Canalis E. Growth hormone, insulin-like growth factors, and the skeleton. Endocr Rev. (2008) 5:535–59. doi: 10.1210/er.2007-0036 PMC2726838 18436706

[2] Mazziotti G Biagioli E Maffezzoni F Spinello M Serra V Maroldi R. Bone turnover, bone mineral density, and fracture risk in acromegaly: A meta-analysis. J Clin Endocrinol Metab. (2015) 2:384–94. doi: 10.1210/jc.2014-2937 25365312

[3] Giustina A. Acromegaly and vertebral fractures: facts and questions. Trends Endocrinol Metab. (2020) 4:274–5. doi: 10.1016/j.tem.2020.01.011 32187523

[4] Chiloiro S Giampietro A Gagliardi I Bondanelli M Veleno M Ambrosio MR. Impact of the diagnostic delay of acromegaly on bone health: data from a real life and long term follow-up experience. Pituitary. (2022) 6:831–41. doi: 10.1007/s11102-022-01266-4 PMC9362053 35922724

[5] Bima C Chiloiro S Mormando M Piacentini S Bracaccia E Giampietro A. Understanding the effect of acromegaly on the human skeleton. Expert Rev Endocrinol Metab. (2016) 3:263–70. doi: 10.1080/17446651.2016.1179108 30058934

[6] de Bakker CMJ Tseng WJ Li Y Zhao H Liu XS. Clinical evaluation of bone strength and fracture risk. Curr Osteoporos Rep. (2017) 1:32–42. doi: 10.1007/s11914-017-0346-3 28185216

[7] Mazziotti G Giustina A. Pituitary diseases and bone. Endocr Rev. (2018) 4:440–88. doi: 10.1210/er.2018-00005 29684108

[8] Chiloiro S Gagliardi I Bianchi A Giampietro A Medici M Allora A. Cholecalciferol use is associated with a decreased risk of incident morphometric vertebral fractures in acromegaly. J Clin Endocrinol Metab. (2023) 109:e58–68. doi: 10.1210/clinem/dgad493 PMC10735684 37606222

[9] Chiloiro S Mirra F Federico D Giampietro A Visconti F Rossi L. The role of growth hormone receptor isoforms and their effects in bone metabolism and skeletal fragility. Protein Pept Lett. (2020) 12:1260–7. doi: 10.2174/0929866527666200616151105 32543356

[10] Mazziotti G Gola M Bianchi A Porcelli T Giampietro A Cimino V. Influence of diabetes mellitus on vertebral fractures in men with acromegaly. Endocrine. (2011) 1:102–8. doi: 10.1007/s12020-011-9486-x 21594681

[11] Mazziotti G Bianchi A Porcelli T Mormando M Maffezzoni F Cristiano A. Vertebral fractures in patients with acromegaly: A 3-year prospective study. J Clin Endocrinol Metab. (2013) 8:3402–10. doi: 10.1210/jc.2013-1460 23771918

[12] Mazziotti G Battista C Maffezzoni F Chiloiro S Ferrante E Prencipe N. Treatment of acromegalic osteopathy in real-life clinical practice: The BAAC (bone active drugs in acromegaly) study. J Clin Endocrinol Metab. (2020) 9:E3285–92. doi: 10.1210/clinem/dgaa363 32511698

[13] Chiloiro S Mormando M Bianchi A Giampietro A Milardi D Bima C. Prevalence of morphometric vertebral fractures in “difficult” patients with acromegaly with different biochemical outcomes after multimodal treatment. Endocrine. (2018) 2:449–53. doi: 10.1007/s12020-017-1391-5 28836162

[14] Chiloiro S Mazziotti G Giampietro A Bianchi A Frara S Mormando M. Effects of pegvisomant and somatostatin receptor ligands on incidence of vertebral fractures in patients with acromegaly. Pituitary. (2018) 3:302–8. doi: 10.1007/s11102-018-0873-7 29397538

[15] Chiloiro S Giampietro A Frara S Bima C Donfrancesco F Fleseriu CM. Effects of pegvisomant and pasireotide LAR on vertebral fractures in acromegaly resistant to first-generation SRLs. J Clin Endocrinol Metab. (2020) 3:4. doi: 10.1210/clinem/dgz054 31613969

[16] Vitali E Palagano E Schiavone ML Mantovani G Sobacchi C Mazziotti G. Direct effects of octreotide on osteoblast cell proliferation and function. J Endocrinol Invest. (2022) 45:1045–57. doi: 10.1007/s40618-022-01740-7 35020172

[17] Vitali E Grasso A Schiavone ML Trivellin G Sobacchi C Mione M. The direct impact of pegvisomant on osteoblast functions and bone development. J Endocrinol Invest. (2023) 47(6):1385–94. doi: 10.1007/s40618-023-02281-3 38159174

[18] Bianchi A Giustina A Cimino V Pola R Angelini F Pontecorvi A. Influence of growth hormone receptor d3 and full-length isoforms on biochemical treatment outcomes in acromegaly. J Clin Endocrinol Metab. (2009) 6:2015–22. doi: 10.1210/jc.2008-1337 19336510

[19] Filopanti M Giavoli C Grottoli S Bianchi A De Marinis L Ghigo E. The exon 3-deleted growth hormone receptor: Molecular and functional characterization and impact on GH/IGF-I axis in physiological and pathological conditions. J Endocrinol Invest. (2011) 11:861–8. doi: 10.1007/BF03346731 22322534

[20] Palizban AA Radmansorry M Bozorgzad M. Exon 3-deleted and full-length growth hormone receptor polymorphism frequencies in an Iranian population. Res Pharm Sci. (2014) 6:489–94. PMC4326986 26339263

[21] Mormando M Nasto LA Bianchi A Mazziotti G Giampietro A Pola E. GH receptor isoforms and skeletal fragility in acromegaly. Eur J Endocrinol. (2014) 2:237–45. doi: 10.1530/EJE-14-0205 24866575

[22] Dos Santos C Essioux L Teinturier C Tauber M Goffin V Bougnères P. A common polymorphism of the growth hormone receptor is associated with increased responsiveness to growth hormone. Nat Genet. (2004) 7:720–4. doi: 10.1038/ng1379 15208626

[23] Falah G Sharvit L Atzmon G. The exon 3-deleted growth hormone receptor (d3GHR) polymorphism—A favorable backdoor mechanism for the GHR function. Int J Mol Sci. (2023) 18:13908. doi: 10.3390/ijms241813908 PMC10531306 37762211

[24] Bernabeu I Alvarez-Escolá C Quinteiro C Lucas T Puig-Domingo M Luque-Ramírez M. The exon 3-deleted growth hormone receptor is associated with better response to pegvisomant therapy in acromegaly. J Clin Endocrinol Metab. (2010) 1:222–9. doi: 10.1210/jc.2009-1630 19850678

[25] Mormando M Chiloiro S Bianchi A Giampietro A Angelini F Tartaglione L. Growth hormone receptor isoforms and fracture risk in adult-onset growth hormone-deficient patients. Clin Endocrinol (Oxf). (2016) 5:717–24. doi: 10.1111/cen.13161 27437620

[26] Schmid C Krayenbuehl PA Bernays RL Zwimpfer C Maly FE Wiesli P. Growth hormone (GH) receptor isoform in acromegaly: lower concentrations of GH but not insulin-like growth factor-1 in patients with a genomic deletion of exon 3 in the GH receptor gene. Clin Chem. (2007) 8:1484–8. doi: 10.1373/clinchem.2007.085712 17573420

[27] Cinar N Dagdelen S Yorgun H Canpolat U Kabakçı G Erbas T. The clinical and cardiometabolic effects of d3-growth hormone receptor polymorphism in acromegaly. Pituitary. (2015) 1:116–25. doi: 10.1007/s11102-014-0564-y 24706164

[28] Mercado M Gonzaílez B Sandoval C Esquenazi Y Mier F Vargas G. Clinical and biochemical impact of the d3 growth hormone receptor genotype in acromegaly. J Clin Endocrinol Metab. (2008) 9:3411–5. doi: 10.1210/jc.2008-0391 18611972

[29] Giustina A Barkhoudarian G Beckers A Ben-Shlomo A Biermasz N Biller B. Multidisciplinary management of acromegaly: A consensus. Rev Endocr Metab Disord. (2020) 4:667–78. doi: 10.1007/s11154-020-09588-z PMC7942783 32914330

[30] Chiloiro S Giampietro A Mirra F Donfrancesco F Tartaglione T Mattogno PP. Pegvisomant and Pasireotide LAR as second line therapy in acromegaly: clinical effectiveness and predictors of response. Eur J Endocrinol. (2021) 2:217–29. doi: 10.1530/EJE-20-0767 33136550

[31] Melmed S Bronstein MD Chanson P Klibanski A Casanueva FF Wass JAH. A Consensus Statement on acromegaly therapeutic outcomes. Nat Rev Endocrinol. (2018) 9:552–61. doi: 10.1038/s41574-018-0058-5 PMC7136157 30050156

[32] Melmed S Casanueva FF Klibanski A Bronstein MD Chanson P Lamberts SW. A consensus on the diagnosis and treatment of acromegaly complications. Pituitary. (2013) 3:294–302. doi: 10.1007/s11102-012-0420-x PMC3730092 22903574

[33] Genant HK Wu CY van Kuijk C Nevitt MC. Vertebral fracture assessment using a semiquantitative technique. J Bone Mineral Res. (2009) 9:1137–48. doi: 10.1002/jbmr.5650080915 8237484

[34] Pantel J Machinis K Sobrier ML Duquesnoy P Goossens M Amselem S. Species-specific Alternative Splice Mimicry at the Growth Hormone Receptor Locus Revealed by the Lineage of Retroelements during Primate Evolution. J Biol Chem. (2000) 25:18664–9. doi: 10.1074/jbc.M001615200 10764769

[35] Franck SE Broer L van der Lely AJ Kamenicky P Bernabéu I Malchiodi E. The effect of the exon-3-deleted growth hormone receptor on pegvisomant-treated acromegaly: A systematic review and meta-analysis. Neuroendocrinology. (2017) 2:131–40. doi: 10.1159/000448844 PMC5637298 27513761

[36] Giustina A Biermasz N Casanueva FF Fleseriu M Mortini P Strasburger C. Consensus on criteria for acromegaly diagnosis and remission. Pituitary. (2024) 27(1):7–22. doi: 10.1007/s11102-023-01360-1 PMC10837217 37923946

[37] Lu M Flanagan JU Langley RJ Hay MP Perry JK. Targeting growth hormone function: strategies and therapeutic applications. Sig Transduct Target Ther. (2019) 4(3):1–11. doi: 10.1038/s41392-019-0036-y PMC6367471 30775002

[38] Wassenaar MJE Biermasz NR Pereira AM van der Klaauw AA Smit JW Roelfsema F. The exon-3 deleted growth hormone receptor polymorphism predisposes to long-term complications of acromegaly. J Clin Endocrinol Metab. (2009) 12:4671–8. doi: 10.1210/jc.2009-1172 19864451

[39] Giustina A. Acromegaly and bone: an update. Endocrinol Metab (Seoul). (2023) 38:655–66. doi: 10.3803/EnM.2023.601 PMC10764988 38164073

[40] Pontes J Madeira M Lima CHA Ogino LL de Paula Paranhos Neto F de Mendonça LMC. Exon 3-deleted growth hormone receptor isoform is not related to worse bone mineral density or microarchitecture or to increased fracture risk in acromegaly. J Endocrinol Invest. (2020) 43:163–71. doi: 10.1007/s40618-019-01096-5 31392573

